# Nanogels as Potential Delivery Vehicles in Improving the Therapeutic Efficacy of Phytopharmaceuticals

**DOI:** 10.3390/polym14194141

**Published:** 2022-10-03

**Authors:** Murtada Taha, Nabil A. Alhakamy, Shadab Md, Mohammad Zaki Ahmad, Md. Rizwanullah, Sana Fatima, Naveed Ahmed, Faisal M. Alyazedi, Shahid Karim, Javed Ahmad

**Affiliations:** 1 Department of Clinical Laboratory Science, Prince Sultan Military College of Health Sciences, Dhahran 31932, Saudi Arabia; 2 Department of Pharmaceutics, Faculty of Pharmacy, King Abdulaziz University, Jeddah 21589, Saudi Arabia; 3 Department of Pharmaceutics, College of Pharmacy, Najran University, Najran 11001, Saudi Arabia; 4 Department of Pharmaceutics, School of Pharmaceutical Education and Research, Jamia Hamdard, New Delhi 110062, Delhi, India; 5 Sufia Unani Medical College Hospital & Research Centre, Bara Chakia, Motihari 845412, Bihar, India; 6 Prince Sultan Military College of Health Sciences, Dhahran 31932, Saudi Arabia; 7 Department of Pharmacology, Faculty of Medicine, King Abdulaziz University, Jeddah 21589, Saudi Arabia

**Keywords:** nanogels, phytopharmaceuticals, biopharmaceutical attributes, nanoemulgel, thixotropy, skin permeation, therapeutic efficacy

## Abstract

Nanogel is a promising drug delivery approach to improve the pharmacokinetics and pharmacodynamic prospect of phytopharmaceuticals. In the present review, phytopharmaceuticals with astonishing therapeutic utilities are being explored. However, their in vivo delivery is challenging, owing to poor biopharmaceutical attributes that impact their drug release profile, skin penetration, and the reach of optimal therapeutic concentrations to the target site. Nanogel and its advanced version in the form of nanoemulgel (oil-in-water nanoemulsion integrated gel matrix) offer better therapeutic prospects than other conventional counterparts for improving the biopharmaceutical attributes and thus therapeutic efficacy of phytopharmaceuticals. Nanoemulgel-loaded phytopharmaceuticals could substantially improve permeation behavior across skin barriers, subsequently enhancing the delivery and therapeutic effectiveness of the bioactive compound. Furthermore, the thixotropic characteristics of polymeric hydrogel utilized in the fabrication of nanogel/nanoemulgel-based drug delivery systems have also imparted improvements in the biopharmaceutical attributes of loaded phytopharmaceuticals. This formulation approach is about to be rife in the coming decades. Thus, the current review throws light on the recent studies demonstrating the role of nanogels in enhancing the delivery of bioactive compounds for treating various disease conditions and the challenges faced in their clinical translation.

## 1. Introduction

Amongst all controlled-release drug delivery systems (such as polymeric nanoparticles and lipid nanoparticles) hydrogels hold unique and substantial importance owing to their thixotropic or sol-gel interconvertible nature. The global sales forecast for hydrogel-based carrier systems is expected to reach a compound annual growth rate (CAGR) of 7.5% from 2021 to 2028 [1]. Hydrogel is one of a kind owing to its characteristic thixotropic property and tunable stiffness (0.5 kPa to 5 MPa) that allow it to be comparable to different soft tissues in the human body [2,3,4]. Hydrogels are three-dimensional polymeric matrices that can imbibe water in large quantities and mimic biological tissues in their swollen state. These attributes of hydrogel make them potential vehicles for encapsulating and delivering bioactive molecules/drugs to the target sites. Hydrogels can very efficiently control the availability of bioactive molecules/drugs to target cells and tissues over a particular range of time. Moreover, the bioactive compound or drug remains protected in the cross-linked network of the hydrogel by the restricted penetration of various degrading enzymes [5].

At present, hydrogels are being explored in various branches of medicine, including pain management, wound healing, cardiology, and oncology [5]. Hydrogels can fall in a wide spectrum of length scales traversing from centimeters to sub-nanometres (nanogels) that dictates the routes by which hydrogels are delivered into the human body [5]. Nanogels serve as vehicles for bioactive molecules/drugs by expediting the absorption or desorption of fluids as a result of environmental changes, including temperature, the presence of enzymes, ionic strength, and pH [6,7,8]. Interestingly, despite nanogels being hydrophilic in nature, they do not get dissolved, rather they swell in the aqueous milieu in vivo attributed to their structured cross-linked network. These structural crosslinks are formed via hydrogen bonding, covalent bonding, Van der Waals interactions, or physical tangling (also called crystallites) [9,10]. This characteristic cross-linked structural network of nanogels allows bioactive molecules/drugs to remain entrapped inside such a three-dimensional matrix for controlled and site-specific release [9,10,11,12,13]. Nonetheless, there are several challenges in encapsulating lipophilic compounds in nanogel matrices, therefore, distinctive approaches are being explored to address these concerns. Upon being swelled up, nanogels become soft and rubbery, possessing low interfacial tension with the aqueous environment and biological fluid [9,13]. Therefore, they tend to simulate body tissues and this property is explicitly used in pharmaceutical drug delivery. Moreover, the elastomeric consistency of the nanogels mitigates mechanical friction between tissues. In this review, the thixotropic property of nanogels is also profoundly discussed. The mechanical strength, thixotropic characteristics, and functionality of nanogel are governed by the physicochemical properties of the polymers it is made up of. The polymeric properties are dictated by various crucial factors including the chemical nature of the monomer, molecular weight, method of polymer synthesis, and macromolecular structure. The characteristic physical structure of the polymer depends on the intensity of the covalent linking, rigidity of bonding, and strength of intermolecular forces inside the polymer chain [9,12,13,14,15,16]. The classification of nanogels is based on various significant properties including the polymeric source (natural, synthetic, or hybrid), structural configuration (semi-crystalline or amorphous), nature of cross-linking (chemical or physical), and electric charge of the network (ionic or neutral) [9,13,14,15,16,17,18,19,20,21]. The distinctive absorption potential of nanogel is because of the presence of special hydrophilic moieties (such as -OH, -CONH, -CONH_2_), in the polymeric components [9,17,18,19,20,21,22,23,24].

**Figure 1:**
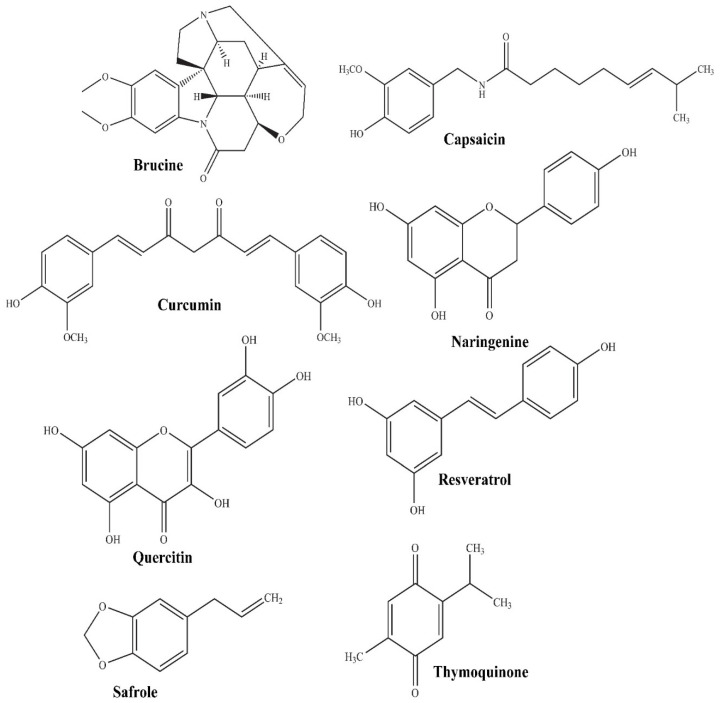
Molecular structure of some promising phytopharmaceuticals.

In the current scenario, herbal or bioactive compounds have gained splendid attention as protective or adjunct therapy in the management of various disorders. The bioactive compounds of natural origin mainly include flavonoids, alkaloids, glycosides, phytosterols, isothiocyanates, saponins, and phenolic acid [25,26]. The phytopharmaceutical terminology covers bioactive compounds (molecular structure illustrated in Figure 1) of phytochemical origin which possess distinctive therapeutic value owing to their potential antioxidant activity, the enrichment of micronutrients, and the ability to preclude the origin of chronic and degenerative ailments [27,28,29].

**Figure 2:**
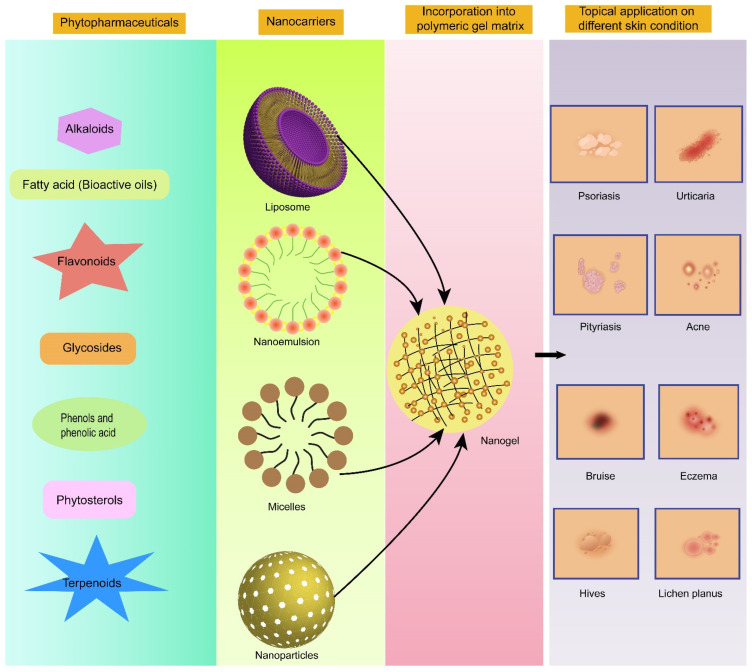
Promising category of phytopharmaceuticals exploiting nanogels as a delivery vehicle for topical application in different skin disorders.

Despite phytopharmaceuticals being substantially capable of treating severe disorders, there are ample numbers of hurdles before they can exert their optimal therapeutic effect. These biopharmaceutical challenges include the low bioavailability of bioactive compounds owing to their low solubility, low permeability, susceptibility to enzymatic degradation, and inability to reach the target sites [30]. Encapsulating the bioactive compounds in nanogels can address these issues very well [31,32,33,34,35,36]. This idea of the delivery of potentially bioactive compounds employing encapsulation in nanogel cross-linked matrices for the treatment of prevalent skin disorders is quite promising (illustrated in Figure 2). More specifically, nanoemulgel has demonstrated astounding benefits for improving the delivery of these bioactive agents with poor biopharmaceutical attributes in comparison to conventional hydrogel formulations.

Nanoemulgel being a drug delivery vehicle consists of oil-in-water nanoemulsion encapsulating therapeutics of lipophilic nature, uniformly dispersed in this drug-loaded emulsion system into a hydrogel-based matrix with the consistency of a semi-solid state [34,35]. The nanoemulgel is capable of improving the dissolution and absorption of encapsulated bioactive compounds in the nanoemulsion system and further controlling and delaying its release due to outside gel embodiment at the target site [33,36]. Therefore, the controlled and sustained release of a therapeutic moiety at the target site could be achieved, along with substantially improved bioavailability and the accomplishment of optimal therapeutic concentration through a nanoemulgel-based drug delivery system. Therefore, in the present manuscript, nanoemulgel preparation that has considerably modified the biopharmaceutical attributes and enhanced the therapeutic efficacy of phytopharmaceuticals is emphasized [37,38,39,40]. In the preceding literature, nanogels have been elucidated as nanocarriers predominantly for the delivery of synthetic drugs/pharmaceuticals. There are a plethora of studies available that have explored the potential of lipid-based carriers such as liposomes, nanoemulsions, and polymeric nanoparticles for the delivery of plant-origin therapeutics including herbal extracts [30,33,34,35,36]. However, limited studies have highlighted the potential of nanogels being a delivery vehicle for bioactive molecules of plant origin.

Thus, the current review throws light on the recent studies demonstrating the role of nanogel/nanoemulgel in improving the delivery of phytopharmaceuticals in various disease conditions and the challenges faced in their clinical translation.

## 2. Thixotropic Property/Rheological Behavior of Nanogels and Its Clinical Significance

The change in the viscosity of nanogel under shear stress (thixotropic nature) finds substantial importance in governing the therapeutic efficacy and performance of incorporated active ingredients via any route of administration, be it topical, oral, mucosal, or ophthalmic. For nanogels to exert efficacious therapeutic action, the non-Newtonian behavior of the thixotropic sol-gel system is highly desirable [41]. This non-Newtonian behavior is dictated by a yield value that is going to structurally break down the solid network system of gel and convert it into a sol state to facilitate plastic flow. The greater this yield value, the stronger the gel network, and the better the therapeutic performance of the nanogel system. Thixotropy is that inclusive characteristic of a nanogel that is going to govern the release behavior, retention efficacy, and systemic bioavailability (in the case of oral preparations) of a therapeutic agent from a loaded nanogel formulation [41]. The thixotropy of a nanogel formulation system depends upon its degree of dispersion and shear history.

The release of a therapeutic agent from a highly interconnected network system is controlled by the influx of water from components of body fluids and the rheological properties of nanoemulgel. The influx rate of body fluids serves as a crucial factor that controls the yield value by swiftly diffusing into the solid matrices of a sol-gel system of nanogel. The yield value of nanogel is further dictated by the intensity of cross-links formed and the hydration level upon the incursion of fluid, which, in turn, will govern the release profile of the encapsulated therapeutic agent [41,42,43]. In a study, it was demonstrated that gel composed of a polyethylene glycol (a Newtonian formulation) matrix spontaneously dissolved upon exposure to simulated saliva, whereas a gel matrix comprising a mixture of Carbopol and polyvinyl-phenol (a non-Newtonian formulation) swelled and formed a viscous barrier to drug release, and the release of a therapeutic agent was governed by the degree of hydration and thixotropic property [41,44]. Such study findings suggest that the improvement of the gelation process or cross-linking by the components of physiological fluids have a significant influence on controlling the release of a loaded therapeutic agent from the thixotropic nanogel formulations in the oral route of drug administration.

**Figure 3:**
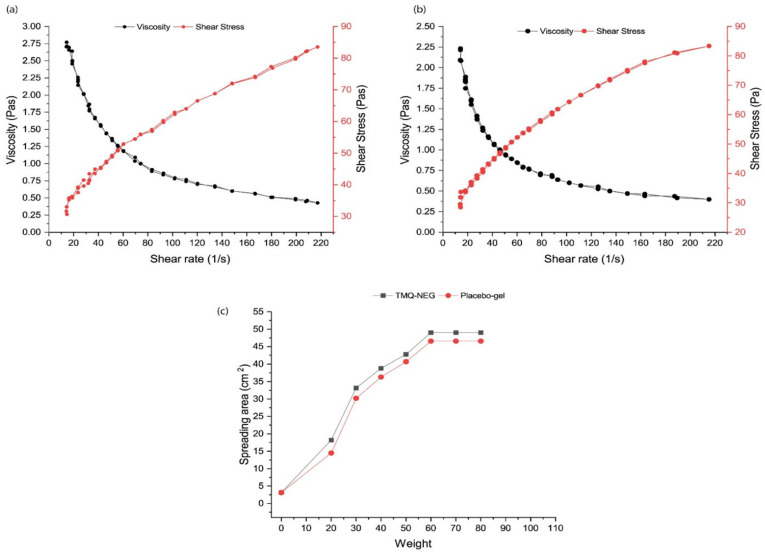
Characteristics of thymoquinone-containing nanoemulgel (TMQ-NEG) system vs. placebo gel. (**a**) Rheology profile of TMQ-NEG system. (**b**) Rheology profile of placebo gel. (**c**) Spreadability behavior of TMQ-NEG vs. placebo gel. Reproduced from [45], Copyright 2021, MDPI.

Importantly, before any topical nanogel formulation is subjected to in vivo evaluation, it is assessed for its rheological profile, which includes spreadability behavior, and retention characteristics over the affected area after application [41]. All of these phenomena are based on attaining a specific yield value, employing manual force that leads to the conversion of gel state to sol state and thus makes nanogel easily extrude out of the container, spread over the application site, and adhere there for long enough to exert a therapeutic effect. Here also, non-Newtonian rheological behavior is highly desirable [41]. The higher the yield value, the greater the force required to spread the nanogel formulation over the application area. In the case of a burn wound, a low yield value is needed for the easy spreadability of the formulation to circumvent the pain of the application of the formulation over the burnt site [41]. Recently, Algahtani et al. designed and developed a thymoquinone-loaded nanoemulgel system for topical application in excision wounds [45]. The developed nanoemulgel system was prepared to utilize Carbopol 940 as a gelling agent at a concentration of 0.5% *w*/*w*. It was observed that the rheological profile of the nanoemulgel system and placebo gel demonstrated similar characteristics (sol-gel behavior) upon the application of shear rate (illustrated in Figure 3a,b). It indicates that the thixotropic characteristics of the Carbopol 940 hydrogel system are not affected by the addition of a phytopharmaceutical-loaded nanoemulsion system and confirms its suitability in the design of nanogels for bioactive therapeutics of plant origin. Furthermore, the spreadability behavior of the Carbopol 940 hydrogel system was not affected by the addition of a phytopharmaceutical-loaded nanoemulsion system (illustrated in Figure 3c) and indicates its suitability for topical application.

Therefore, in the case of topically applied gel formulations, the thixotropy property also plays an ultimate key role in governing the biopharmaceutical performance of nanogel. In the subsequent section, the biopharmaceutical characteristics of nanogel loaded with phytopharmaceuticals as therapeutic agents have been investigated in recent studies and are being discussed in detail.

## 3. Overview and Biopharmaceutical Characteristics of Phytopharmaceutical-Containing Nanogels

Plant species serve as a massive reservoir of medicinally active compounds, whose usefulness and functionality are untapped until today [46]. The obstacles encountered with the administration of phytochemical compounds for treating ailments include limited stability, poor aqueous solubility, and low bioavailability, which restrict their therapeutic efficacy [47,48]. Recently, those investigations are gaining momentum that explores the use of herbal compounds and oils for the treatment of various ailments. The introduction of nanotechnology in the arena of herbal bioactive compound delivery has renovated the results of therapeutic interventions [48]. The loading of a bioactive compound into the nanosized preparations certainly improves its physicochemical and pharmacokinetic attributes, subsequently resulting in improved therapeutic prospects [48]. Nanogels present a promising formulation approach in this context for improving the solubility, penetrability, stability, pharmacokinetic fate, and overall therapeutic efficacy of bioactive compounds. The specific range of sizes and shapes of nanoparticulate delivery vehicles in nanogel systems allows the bioactive compound to attain substantial targetability in the body and the interconvertibility of sol-gel states results in the prolonged interaction of the nanogel system with the diseased tissue/application site [47,48]. The phytochemical compounds of different classes (alkaloids, phenols, fatty acids, and terpenoids) have been exploited to improve their biopharmaceutical characteristics through the nanogel formulation approach to accomplish optimal therapeutic effects, as discussed hereunder.

### 3.1. Phytopharmaceuticals of Alkaloids Category

The bioactive compounds that fall under the alkaloids category present a plethora of therapeutically active agents. Alkaloids are heterocyclic nitrogenous compounds and can be classified as pyrrolidines, quinolines, phenanthrenes, purines, or imidazoles based on their molecular structure [48,49]. Amongst various alkaloids of different classes, the most explored bioactive compounds that have utilized nanogels as a delivery vehicle to enhance their biopharmaceutical performance include berberine, capsaicin, and brucine.

#### 3.1.1. Capsaicin

Capsaicin has been studied for its therapeutic prospects in various diseases, including several carcinomas, rheumatoid arthritis, and diabetic neuropathy, and studies have established it to be extremely effective as a phytopharmaceutical [50,51]. However, its short half-life (7.06 min), extensive first-pass metabolism, pungency, and patient non-compliance hamper its clinical applicability for treating the aforementioned disorders. To overcome these limitations, attempts were made to encapsulate capsaicin in nanogel to overcome the hurdles in attaining its optimal therapeutic effect. The nanogel formulation of capsaicin resulted in substantially enhanced skin permeation as compared to the conventional gel system. Therefore, employing the encapsulation of capsaicin in nanogel formulations, its physicochemical properties such as size and shape were modulated in a way that facilitated its improved transdermal administration in patients with diabetic neuropathy [50].

#### 3.1.2. Berberine

Berberine, an isoquinoline alkaloid, possesses a huge amount of therapeutic activities, including a reduction in blood sugar, improvement in cardiovascular disorders, and significant antimicrobial activity against several microbes [52]. However, there are significant biopharmaceutical challenges (poor permeability, low gastrointestinal stability, limited aqueous solubility) with its delivery, which can supposedly be addressed by employing a nanogel formulation approach. Amato et al. have reported that 50% of loaded berberine is released from the nanogel system within 45 min [53]. In another investigation, Xu et al. reported improvement in the biopharmaceutical characteristics of berberine through designing a nanoemulsion system for oral application [54]. This developed nanoemulsion system could be converted into a nanoemulgel system and would be a promising approach to improving the biopharmaceutical characteristics of berberine after topical application.

#### 3.1.3. Brucine

Interestingly, Brucine possesses anti-inflammatory and anti-nociceptive activity that can be explored to relieve arthritis and traumatic pain. However, its clinical utility is considerably hindered by its low aqueous solubility, gastrointestinal complications, and systemic toxicity that might occur after its oral administration. Brucine nanoemulgel with specific particle size, spreadability, and viscosity altogether improved its release and permeation profile. The ex vivo drug permeation of the Brucine nanoemulgel formulation system on rat skin demonstrated significantly boosted permeation and the markedly increased transdermal flux of Brucine nanogel as compared to Brucine suspension [55]. The results of this investigation may provide an approach to improving the biopharmaceutical characteristics of natural bioactive products utilizing a nanoemulgel formulation system.

### 3.2. Phytopharmaceuticals of the Fatty Acids Category

Fatty acids (saturated and unsaturated) represent an enormous source of therapeutically functional compounds whose usage is highly encouraged by exploiting the nanogel formulation approach. Based on the results of solubility and compatibility studies, the priority of researchers is always to go for an oil of herbal origin into which to load the lipophilic therapeutically active agent [56]. Oleic acid is being widely explored as a formulation excipient in many nanoparticulate formulations [57]. Other than olive oil, oil from *Nigella sativa* seeds is also being investigated for therapeutic application employing the nanogel formulation approach. Recently, Algahtani et al. have designed and developed a nanoemulgel system containing the oil of *Nigella sativa* seeds for topical application on excision wounds [45]. The nanoemulsion in the nanoemulgel system may provide greater surface area for the deeper skin penetration of fatty acid oil in a form of nano oil droplets after topical application in different disease conditions.

### 3.3. Phytopharmaceuticals of the Phenolic Category and Related Compounds

The phenolics class of compounds exhibits innumerable therapeutic properties [58]. Phenolic compounds comprise complex molecules including coumarins, flavonoids, tannins, anthraquinones, and stilbenes [59].

#### 3.3.1. Rutin

Rutin is one of the widely explored bioactive compounds of this class in various nanoparticle formulations attributed to its significant antioxidant, neuroprotective, cardioprotective, and anticancer properties [48]. Its chitosan/poly (acrylic acid) nanogel formulation was formulated by Radwan and Ali and has demonstrated noteworthy improvement in the percentage of rutin release at alkaline pH (7.4) compared to the acidic pH (2.0) of the release media [60]. This system was developed for oral administration. It is anticipated that if rutin is encapsulated in nanoemulgel it would considerably improve its biopharmaceutical characteristics such as drug release and thus permeability across biological barriers owing to the nano-range globule size and the desirable surface morphology of nanoemulgel systems developed for topical application.

#### 3.3.2. Hesperetin, α-Mangostin

Another important bioactive compound is hesperetin, which is a flavanone-glycoside found in citrus fruits and possesses potent therapeutic activities, including lipid regulating, anti-inflammatory, and anticancer activities [61]. α-mangostin, belonging to this class of compound, has been reported to possess antibacterial and anti-inflammatory activity [62,63]. All these compounds suffer limited aqueous solubility-related problems which lead to poor biopharmaceutical performance and low therapeutic efficacy compared to the formulation design developed without the utilization of nanotechnology [64].

#### 3.3.3. Resveratrol, Curcumin

Resveratrol is another compound of this class that possesses potential anti-inflammatory, anticarcinogenic, cardioprotective, and neuroprotective activities [65]. However, its inability to cross the skin membrane owing to poor aqueous solubility is the major hurdle in attaining its optimum therapeutic effect through the topical route of drug administration. Another bioactive compound that is of great interest to researchers is curcumin; however, it also has major pharmaceutical setbacks due to poor water solubility and limited stability [66,67,68,69,70]. Therefore, the poor skin penetration of polyphenols and their confinement to the stratum corneum layer were reported as one of the major issues in their topical and transdermal administration [65].

In the current scenario, a lot of the focus of scientists has shifted towards the encapsulation of phenolic bioactive compounds in novel nanogel, intending to improve their target reach and accumulation [65]. In an interesting study, the delivery of resveratrol and curcumin encapsulated nanogel carriers was shown to significantly improve their skin-delivery and wound-healing potential [65]. The improved skin delivery of these phenolic compounds was attributed to the improved physicochemical properties of resveratrol and curcumin employing nanoemulgel formulations. The nanometer-sized system assured efficient deposition into the deep layers of the skin. It has been established in various studies that particles smaller than ≤300 nm could reside in the deep skin layers [71], and therefore nanometric size range could facilitate the deeper skin permeation of polyphenols through the utilization of nanogel as a delivery vehicle. Moreover, the prepared negatively charged nanoparticles also contributed to absorption across skin layers. The sufficient viscosity of the nanoemulgel is also required for assuring the sufficiently delayed retention and adhesivity of the nanogel formulation. This is how these biopharmaceutical characteristics, namely particle size, surface charge, and viscosity, could contribute significantly to improving the delivery of polyphenols into the deeper skin layers.

#### 3.3.4. Naringenin, Quercetin

In an interesting study finding, naringenin nanoemulgel was designed and characterized for topical application in chronic wound conditions with the intent to improve drug release and skin absorption and limited the biopharmaceutical fate of naringenin. The physicochemical properties of naringenin nanoemulgel, namely globule size, surface charge, viscosity, mucoadhesive property, and spreadability, were critically optimized. The results of in vitro release studies demonstrated remarkable improvement in naringenin release from the formulated nanoemulgel, attributed to the nanometric size of the oil globules (145.58 ±12.5) of the dispersed phase [72]. Therefore, by duly modifying the physicochemical properties of nanoemulgel, a significant modification in the release and thus dissolution profile can be brought about by employing a nanogel formulation. Furthermore, in another study, a nanoemulgel of quercetin, (a potent antimicrobial and anti-inflammatory agent) was formulated to improve its solubility and bioavailability for the treatment of periodontitis. The formulated nanoemulgel exhibited a significantly increased release of quercetin (92.4%) as compared to that of pure quercetin-loaded gel (<3% release) [73]. These study findings are clear evidence of the enhancement of the biopharmaceutical prospects of a phenolic compound employing a nanogel formulation.

### 3.4. Phytopharmaceuticals of Terpenoids Category

Terpenoids, structurally built of isoprene blocks, are the major plant-derived secondary metabolites owning an assortment of pharmacological activities [74,75].

#### Thymoquinone

**Figure 4:**
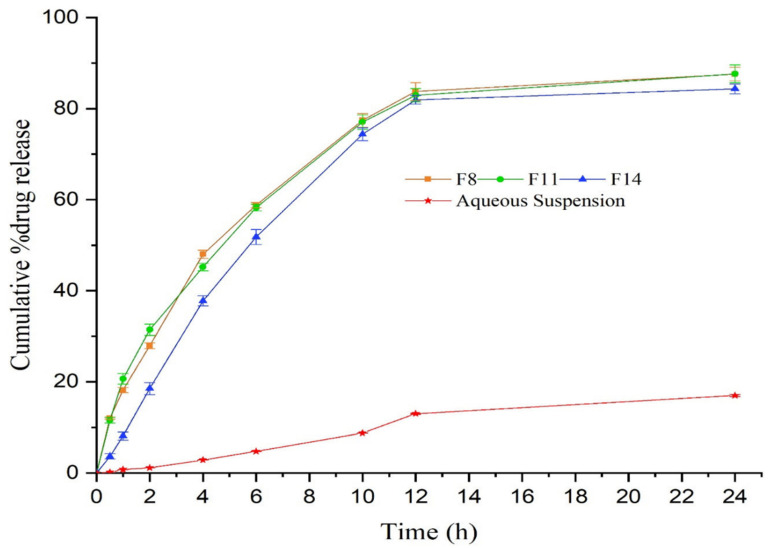
In vitro release of thymoquinone from nanoformulations differing in composition (F8, F11, and F14) vs. the aqueous suspension of thymoquinone carried out through the dialysis bag technique. Reproduced from [45], Copyright 2021, MDPI.

Thymoquinone is a potent and efficacious terpenoid with substantial therapeutic activity against multiple ailments. Nonetheless, its very low aqueous solubility and inadequate skin permeability limit its clinical applicability [76]. A nanoemulgel system was designed and characterized to improve the delivery of thymoquinone [45]. The size of formulated nanodroplets was less than 100 nm, which lead to notable improvement in the release profile of thymoquinone from the nanoemulgel system (Illustrated in Figure 4).

Moreover, the developed nanoemulgel system of thymoquinone has pseudoplastic behavior with thixotropic properties that greatly improve the topical efficacy of thymoquinone. It was demonstrated in the study outcome that the thymoquinone nanoemulgel system exhibited significantly augmented skin penetrability and deposition characteristics post-topical administration in comparison to the conventional hydrogel system. One of the reasons for the greatly improved skin penetration is the presence of the nanoemulsion-loaded thymoquinone system in gel, as nanoemulsions provide an enormous surface area for the better penetration of therapeutic agents into the pilosebaceous region, resulting in better efficacy [45].

**Figure 5:**
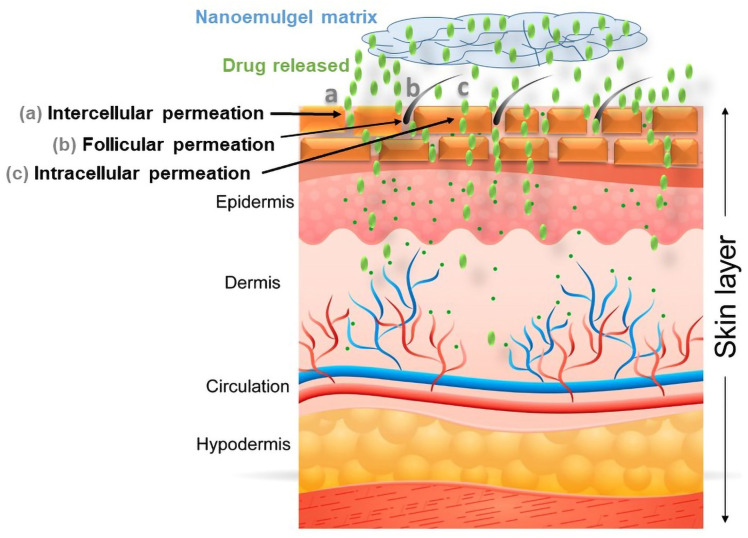
Illustration highlights the improved permeation of the bioactive compound (through intercellular, follicular, and intracellular permeation) to the deeper layer of skin utilizing a nanoemulgel system as a delivery vehicle.

From the above studies, it can be concluded that a nanoemulsion-based hydrogel system (nanoemulgel) could greatly improve the drug release profiles, absorption (across deeper skin layers), retention in the targeted area, and overall biopharmaceutical fate of encapsulated bioactive compounds (illustrated in Figure 5), summarized in Table 1.

The therapeutic performance of nanogel-loaded phytopharmaceuticals investigated in a recent study is reviewed and discussed in detail in the subsequent section for different disease conditions.

## 4. Current Trend and Future Prospects of Phytopharmaceutical-Containing Nanoemulgel for Treating Acute/Chronic Disorders

The bioactive compounds loaded in nanogel/nanoemulgel preparations are being actively explored for pre-clinical evaluations. The promising study findings of the in vitro release and ex vivo skin penetration of nanogel formulations containing bioactive compounds, as discussed in the earlier section, compel researchers to go further for the pre-clinical evaluation of these formulations for different pharmacodynamic activities in a suitable animal model.

### 4.1. Anti-Inflammatory and Anti-Nociceptive Activity

For instance, when Brucine-loaded nanoemulgel showed markedly enhanced skin permeation (as discussed in an earlier section), it was further evaluated for anti-inflammatory and anti-nociceptive activity [55]. For assessing anti-inflammatory activity, the carrageenan-induced rat hind paw edema method was utilized, and for evaluating anti-nociceptive activity, the hot plate method and acetic acid-induced writhing test were employed for Brucine nanoemulgel and comparison formulations in male BALB/c mice. The study outcome revealed the efficient anti-inflammatory and anti-nociceptive potential of Brucine-loaded nanoemulgel in comparison to other prepared formulations and established nanoemulgel as a potential delivery vehicle for expediting the anti-inflammatory and anti-nociceptive actions of Brucine [55]. In another study, the anti-inflammatory potential of curcumin and emu oil by incorporating both in a nanogel system was evaluated in in vivo models by topical route [77]. The anti-inflammatory efficacy was evaluated in carrageenan-induced paw edema and Freund’s complete adjuvant (FCA)-induced arthritic rat model. The in vivo evaluation demonstrated significant improvement in anti-inflammatory activity with the nanogel formulation of curcumin and emu oil [77]. Therefore, the nanogel formulation system markedly improved the therapeutic potential of incorporated oils in rheumatoid arthritis indications. As it is known that mangosteen rind possesses remarkable anti-inflammatory activity, its nanoemulgel was therefore investigated for the same in carrageenan-induced laboratory mice [78]. The in vivo results revealed that mangosteen rind-loaded nanoemulgel demonstrated significant inflammatory inhibition (*p* < 0.05) compared to conventional mangosteen rind gel.

### 4.2. Wound Healing Activity

In another study, the nanoemulgel system of thymoquinone was shown to exhibit markedly enhanced and early healing effects in wounded Wistar rats as compared to a conventional hydrogel of thymoquinone. The outcome of this investigation confirms that the topical delivery of thymoquinone was substantially improved through incorporation into the nanoemulgel system, which accelerated the process of wound healing [45].

**Figure 6:**
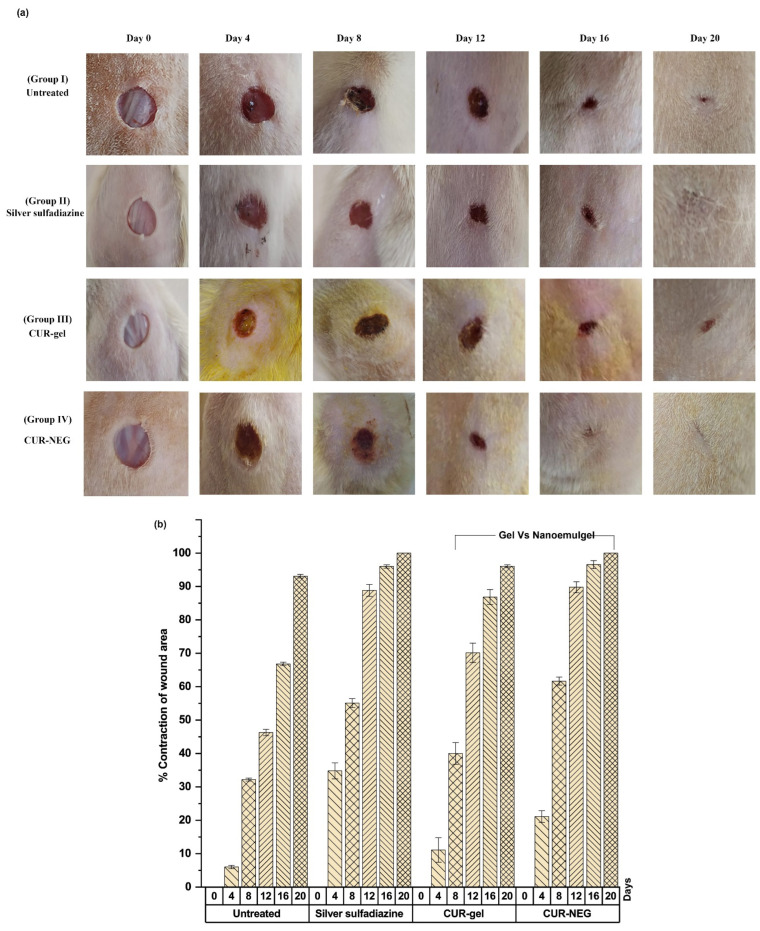
Illustration highlights improved wound healing efficacy of curcumin utilizing nanoemulgel as a delivery vehicle in Wistar rats. (**a**) Wound healing efficacy of curcumin nanoemulgel compared to the control, a marketed product, and curcumin-containing conventional gel. (**b**) Percentage of contraction of wound area of curcumin nanoemulgel compared to the control, a marketed product, and curcumin-containing conventional gel. Reproduced from [79], MDPI, 2021.

Interestingly, the specific physicochemical properties of nanoemulgel, including retaining moisture, exudate absorption, and gas permeability, provide it huge supremacy as a drug delivery vehicle in the case of wound healing applications. In this context, a curcumin-loaded nanoemulgel system was designed and developed for assessing its wound healing potential [79]. The developed curcumin nanoemulgel exhibited thixotropic rheological behavior and a significant (*p* < 0.05) increase in skin penetrability characteristics compared to the curcumin hydrogel system. Importantly, the in vivo wound healing efficacy study and the histological examination of healed tissue specimens revealed that the nanoemulgel-based approach contributed significantly to improving the biopharmaceutical attributes and thus wound healing efficacy of curcumin (Illustrated in Figure 6).

### 4.3. Antimicrobial and Anticancer Activity

Researchers have explored the antimicrobial and anticancer effects of coriander oil incorporated in a nanoemulgel formulation. Encouraging results were obtained with the nanoemulgel for coriander oil’s antimicrobial potential against different types of bacteria [80]. Furthermore, the results of the anticancer activity of coriander oil nanoemulgel were found to be remarkably boosted when applying the prepared formulation to human breast cancer cells (MCF-7), hepatocellular carcinoma cells (Hep3B), and human cervical epithelioid carcinoma cells (HeLa), which signifies that the nanoemulgel substantially enhanced the anticancer effects of coriander oil [80]. Therefore, a remarkable formulation benefit of nanoemulgels as a drug delivery system is that it significantly enhances the therapeutic efficacy of loaded bioactive compounds. In another study, a local dental nanoemulgel formulation of *Nigella sativa* oil was formulated for the treatment of periodontal diseases that showed notably improved antimicrobial activity compared to plain *Nigella sativa* oil [81]. Furthermore, Safrole is a natural compound that possesses remarkable biological activities. Safrole oil has potential antimicrobial and anticancer activities, and these activities were shown to be improved with safrole nanoemulgel formulation [82]. In a study, the antimicrobial activity of the safrole oil and safrole nanoemulgel were tested and their cytotoxic potential was assessed against Hep3B cancer cell lines using the MTS assay. The study outcome revealed that the anticancer and antioxidant activities of safrole could be substantially improved by employing its nanoemulgel formulations.

### 4.4. Antipsoriatic Activity

The nanoemulgel formulation of curcumin was also investigated for treating psoriasis and the study outcome suggested that curcumin nanoemulgel exhibited quicker and early healing in psoriatic mice compared to curcumin alone [83]. The study findings are strongly indicative of curcumin nanoemulgel as a promising and potential candidate for the better long-term management of psoriasis.

Overall, the above discussion of recent studies is clear evidence of the significant contribution of nanogel formulations as a delivery vehicle in enhancing the biopharmaceutical fate and thus therapeutic efficacy of loaded phytopharmaceuticals (summarized in Table 2). However, there are several hurdles in the clinical translation of bioactive compound-loaded nanogels/nanoemulgels that are discussed in the subsequent section.

## 5. Hurdles in Clinical Translation of Phytopharmaceutical-Containing Nanogels

The ultimate goal of emphasizing the benefits of phytopharmaceutical-loaded nanogel is their clinical translation. However, the number of phytopharmaceutical-loaded nanogels that have entered the clinic is limited. Various hurdles impede the commercialization of phytopharmaceutical-loaded nanogels for different indications. The significant challenges in their clinical translation include nanogel fabrication at a commercial scale, storage stability, regulatory complexity, and cost-effectiveness. The high-water content of nanogel presents a great difficulty in their terminal sterilization, and sterility must therefore be typically validated for all source materials and fabrication processes. There are other complications related to the degradation of nanogels, therefore the nanogel must be dehydrated after fabrication to prevent premature degradation during storage [84,85]. The dehydration process must ensure that the nanogel structure and the bioactivity of the phytopharmaceuticals remain intact during treatment. In case nanogels are maintained in a hydrated state during storage, the storage conditions should minimize water evaporation and preclude pre-exposure to any medium that causes the undesirable loss of the phytopharmaceuticals. The impact of the storage conditions must be carefully examined on phytopharmaceutical stability [84].

The fabrication of nanoemulgels is cost-consuming; for instance, particle-size reduction for attaining a nano-size range requires high energy and specialized devices [85]. At the industrial scale, the production of a nanoemulgel involves high-energy methods accompanied by high-pressure homogenizers, which are expensive and demand maintenance costs. Moreover, microfluidization and ultrasonication techniques are the most commonly used techniques for achieving size in nano-dimensions, but they are also expensive and require an extra start-up cost compared to conventional gel formulations, which leads to a delay in achieving its commercialization scalability [84,85]. Although, the design of nanoemulsions through spontaneous emulsification (low-energy emulsification) techniques and conversion into nanogel formulation utilizing a gelling agent as a cost-effective nano product is also a trend.

There are considerable regulatory concerns, and the cost of commercialization is often a notable hurdle present in the clinical translation of phytopharmaceutical-loaded nanogels. The nanogel encapsulating a bioactive compound is considered a combination product, and therefore its regulatory approval process can take 7–10 years [84]. As the duration of patent protection is limited, a longer approval time can limit the commercial viability of nanogels encapsulating phytopharmaceuticals. Moreover, the cost of developing nanogel from bench to bedside is estimated to be high, which provides a significant setback to commercialization [85]. On top of that, phytopharmaceuticals, including safrole, are very costly, therefore the development of the nanogel preparations of such bioactive compounds is a costly affair that discourages the product development and clinical translation of phytopharmaceutical-loaded nanogels. Researchers need to plug these loopholes for successful clinical translation, expediting the commercialization process of phytopharmaceutical-based nanogels by exploiting cost-effective technology.

## 6. Conclusions

The present review concludes that nanogel is a potential delivery system for enhancing the biopharmaceutical and therapeutic prospects of phytopharmaceuticals. The thixotropic characteristics of nanogel have demonstrated their significant role in enhancing the biopharmaceutical attributes of phytopharmaceutical-loaded preparations. Modification in nanogels such as nanoemulgel, which consists of a nanoemulsion incorporated into a hydrogel matrix, can efficiently resolve various problems related to phytopharmaceutical delivery, including the drug-release profile, physical/chemical stability, skin penetrability to a deeper layer, and targeted release. Furthermore, hydrogels are also utilized to fabricate polyphenol-based composite hydrogel systems for use in the field of tissue engineering and the design of hybrid hydrogel systems exploiting stimuli-responsive smart materials for imparting multifunctionality to the hydrogels [86,87,88]. The pre-clinical investigations of nanoemulgel have demonstrated substantially improved biopharmaceutical fate and therapeutic responses of encapsulated phytopharmaceuticals, as discussed in detail in this review. Therefore, nanoemulgel represented a potential efficacious delivery approach for improving the therapeutic prospect of phytopharmaceuticals and its clinical translation-related hurdles.

## Figures and Tables

**Table 1:** Physicochemical characteristics and biopharmaceutical performance of phytopharmaceutical-containing nanoemulgel systems.

Active Agent	Gelling Agent	Physical Characteristics	Biopharmaceutical Performance	Ref.
Thymoquinone	Carbopol 940 (0.5% *w*/*w*)	Mean droplet size of incorporated nanoemulsion <100 nm, pseudoplastic behavior with thixotropic properties.	Significant increase (*p* < 0.05) in skin permeability and deposition profile.	[45]
Capsaicin	Carbopol 940 (1% *w*/*w*)	Pseudoplastic behavior of the nanoemulgel and decrease in viscosity with an increase in the shear rate.	Nanoemulgel revealed a four-fold improvement in capsaicin’s cumulative permeation in comparison to the conventional gel.	[50]
Brucine	Sodium carboxymethylcellulose (1% *w*/*w*)	Higher drug release from nanoemulgel formulation compared to emulgel formulation.	Skin permeation of Brucine through rat skin was found to be greatly improved in the case of the nanoemulgel system compared to the drug solution.	[55]
Resveratrol and Curcumin	Carbopol (2.5% *w*/*w*)	Viscosity of nanoemulgel was found to be 16,020 ± 30.87 cp and particle size of nanoemulsions was 180 ± 5.20 nm.	Significant retention of the phytopharmaceuticals in the skin through nanoemulgels, reaching about 60% of the applied dose, observed after 48 h.	[65]
Naringenin	Carbopol 934 and Carbopol 940 (1%, 1.5%, 2% *w*/*v*)	Uniform dispersion (PDI, 0.452 ± 0.03) of the nanometric globules (145.58 ± 12.5) of the dispersed phase and good spreadability.	Improved and sustained release up to a maximum of 74.62 ± 4.54% from the developed nanoemulgel within the time frame of 24 h.	[72]
Quercetin	Poloxamer (23% *w*/*v*)	Viscosity was 408.3 ± 0.5 cPs at 26 ± 0.5 °C and 30,647.5 ± 0.3 cPs at 37 ± 0.5 °C, demonstrating the sol-gel nature of the formulation.	The developed nanoemulgel exhibited a significant release of 92.4% of quercetinat the end of 6 h, as compared to that of pure quercetin-loaded gel (<3% release).	[73]

**Table 2:** Contemporary research highlighting the in vivo improvement in the therapeutic efficacy of phytopharmaceutical-containing nanoemulgel.

Phytopharmaceutical	In Vivo Model	Application	Therapeutic Outcome	Ref.
Thymoquinone	Wistar rats	Topical	Nanoemulgel formulation showed quicker and early healing in wounded rats compared to the conventional hydrogel system.	[45]
Capsaicin	Swiss-Webster mice	Transdermal	Improvement in anti-nociceptive properties was observed in the treated diabetic mice.	[50]
Brucine	BALB/c mice; Carrageenan-induced rat hind paw edema method.	Topical	Improved anti-inflammatory and anti-nociceptive activity of Brucine-loaded nanoemulgel.	[55]
Resveratrol and curcumin	Wistar rats	Topical	Augmented the burn-healing potential of the nutraceutical combination nanoemulgel.	[65]
Curcumin and emu oil	Carrageenan-induced paw edema and FCA-induced arthritic rat model	Topical	Significant improvement in anti-inflammatory activity with nanoemulgel formulations compared to pure curcumin.	[77]
Mangosteen rind	Mice	Topical	Nanoemulgels of mangosteen rind fraction demonstrated potential anti-inflammatory activity.	[78]
Curcumin	Albino rats	Topical	Improved the wound-healing activity of curcumin compared to the conventional gel formulation of curcumin.	[79]
Coriander oil	Klebsiella pneumoniae, Escherichia coli, Staphylococcus aureus, Pseudomonas aeruginosa. Cancer cell line (MCF-7, Hep3B)	In vitro	Marked antimicrobial and anticancer activities, as compared to those in crude oil and positive control medications.	[80]
*Nigella sativa* oil	Staphylococcus aureus strain (ATCC 29213)	In vitro	Markedly improved antimicrobial activity compared to the plain *Nigella sativa* oil.	[81]
Safrole	Hep3B cancer cell line	In vitro	Improved antimicrobial and anticancer activities by means of safrole nanoemulgel.	[82]
Curcumin	BALB/c mice; Psoriasis induced by topical application of imiquimod cream	Topical	Nanoemulgel system showed quicker and early healing in psoriatic mice compared to curcumin gel.	[83]

## Data Availability

Data sharing not applicable.
